# Integrated risk stratification for ICI-associated myocarditis: a baseline hematological profile and a combined ECG and enzymatic signature at onset

**DOI:** 10.3389/fimmu.2026.1762144

**Published:** 2026-03-31

**Authors:** Zhuoling Zheng, Chunmei Dai, Qian Wu, Jingwen Xie, Min Gao, Xiaoyan Li

**Affiliations:** 1 Department of Pharmacy, The Sixth Affiliated Hospital, Sun Yat-Sen University, Guangzhou, Guangdong, China; 2 Biomedical Innovation Center, The Sixth Affiliated Hospital, Sun Yat-sen University, Guangzhou, Guangdong, China; 3 School of Pharmacy & Clinical Pharmacy (School of Integrated Pharmacy), Guangdong Pharmaceutical University, Guangzhou, Guangdong, China

**Keywords:** creatine kinase, electrocardiogram, immune checkpoint inhibitor, myocarditis, risk stratification

## Abstract

**Background:**

Immune checkpoint inhibitor-associated myocarditis (ICI-associated myocarditis) is a rare but fatal immune-related adverse event. Early identification of high-risk patients remains challenging. This study aimed to identify risk factors and develop models for predicting both the occurrence and severity of ICI-associated myocarditis.

**Methods:**

This retrospective unmatched case-control study stratified patients receiving ICIs into ICI-associated myocarditis (stratified into mild and severe subgroups) and controls. Comparative analysis of baseline and onset-phase data was performed, with logistic regression used to identify risk factors for the development of ICI-associated myocarditis and the severe myocarditis.

**Results:**

In this cohort of 196 patients (98 myocarditis cases *vs.* 98 controls), a two-tiered risk stratification was established. Patients with myocarditis were further stratified into mild (n=71) and severe (n=27) subgroups. For predicting the occurrence of ICI-associated myocarditis, a baseline model incorporating elevated eosinophil ratio, reduced lymphocyte ratio, and elevated myoglobin demonstrated an area under the ROC curve (AUC) of 0.699 (95% CI, 0.626-0.772, *P* < 0.001). Upon onset, for predicting severe myocarditis, a model combining electrocardiographic abnormalities (T-wave changes, bundle branch blocks) and marked CK elevation (>10× ULN) achieved a higher AUC of 0.769 (95% CI, 0.655-0.882, *P* < 0.0001). Severe cases presented significantly earlier than mild cases (33 *vs*. 63 days, *P* = 0.043) and had higher rates of symptoms and concurrent immune-related adverse events.

**Conclusion:**

A baseline profile of elevated eosinophil ratio, reduced lymphocyte ratio, and elevated myoglobin collectively may help identify patients at risk for ICI-associated myocarditis. At myocarditis onset, a combination of specific electrocardiographic abnormalities and marked CK elevation may predict severe cases. These findings suggest a two-stage approach for early risk stratification and targeted management.

## 1 Introduction

Immune checkpoint inhibitor (ICI) has become a standard treatment in oncology. By activating the host immune system against tumors, they have significantly improved clinical outcomes across various malignancies (1). However, this immune activation can also lead to immune-related adverse events (irAEs) resulting from off-target inflammation in normal tissues (2–5). These irAEs are mediated by activated T cells (6).

Among these irAEs, cardiovascular toxicities, particularly ICI-associated myocarditis, are rare but represent the most severe and life-threatening complications (7, 8). The incidence of cardiovascular events in patients receiving ICIs is reportedly 11% higher than in those not undergoing such treatment, with myocarditis being the predominant manifestation (9). Although the overall incidence of ICI-associated myocarditis is low (10, 11), it carries an high mortality rate of approximately 46% (12), underscoring a critical risk in an otherwise effective therapy.

Recent studies have identified several risk factors for ICI-associated myocarditis. A validated prognostic risk score includes active thymoma, cardio-muscular symptoms, low QRS voltage, left ventricular ejection fraction <50%, and troponin elevation (13). Electrocardiographic (ECG) abnormalities such as complete heart block, prolonged QRS duration, and dynamic ST-T changes (14–17), as well as laboratory markers including absolute lymphocyte count, B-type natriuretic peptide, troponin, lactate dehydrogenase and lactate dehydrogenase-to-albumin ratio (14, 16, 18, 19), have also been associated with ICI-associated myocarditis. Additional risk factors include dual ICI therapy (20), pre-existing autoimmune diseases (21), and thymoma (13).

Despite these advances, challenges remain. First, although various risk factors have been identified, well-defined baseline risk predictors for ICI-associated myocarditis are still limited, and existing models often rely on variables not routinely available in all settings. Second, although major oncology societies provide guidelines (22, 23) for the general management of irAEs, the specific criteria for grading ICI-associated myocarditis remain ambiguous. While the Common Terminology Criteria for Adverse Events (CTCAE) (24) is widely used, it does not clearly delineate distinct clinical features, such as specific patterns in cardiac injury biomarkers and ECG parameters that characterize different severity grades.

To address these gaps, this real-world, retrospective case-control study was designed with two primary objectives: first, to identify baseline risk factors for ICI-associated myocarditis through comparison with controls; and second, to characterize severe myocarditis by systematically comparing symptomatology, ECG parameters, and cardiac injury biomarker patterns between mild and severe cases at presentation. The findings aim to establish a data-driven foundation for early risk stratification and severity assessment.

## 2 Materials and methods

### 2.1 Study design

**Figure 1:**
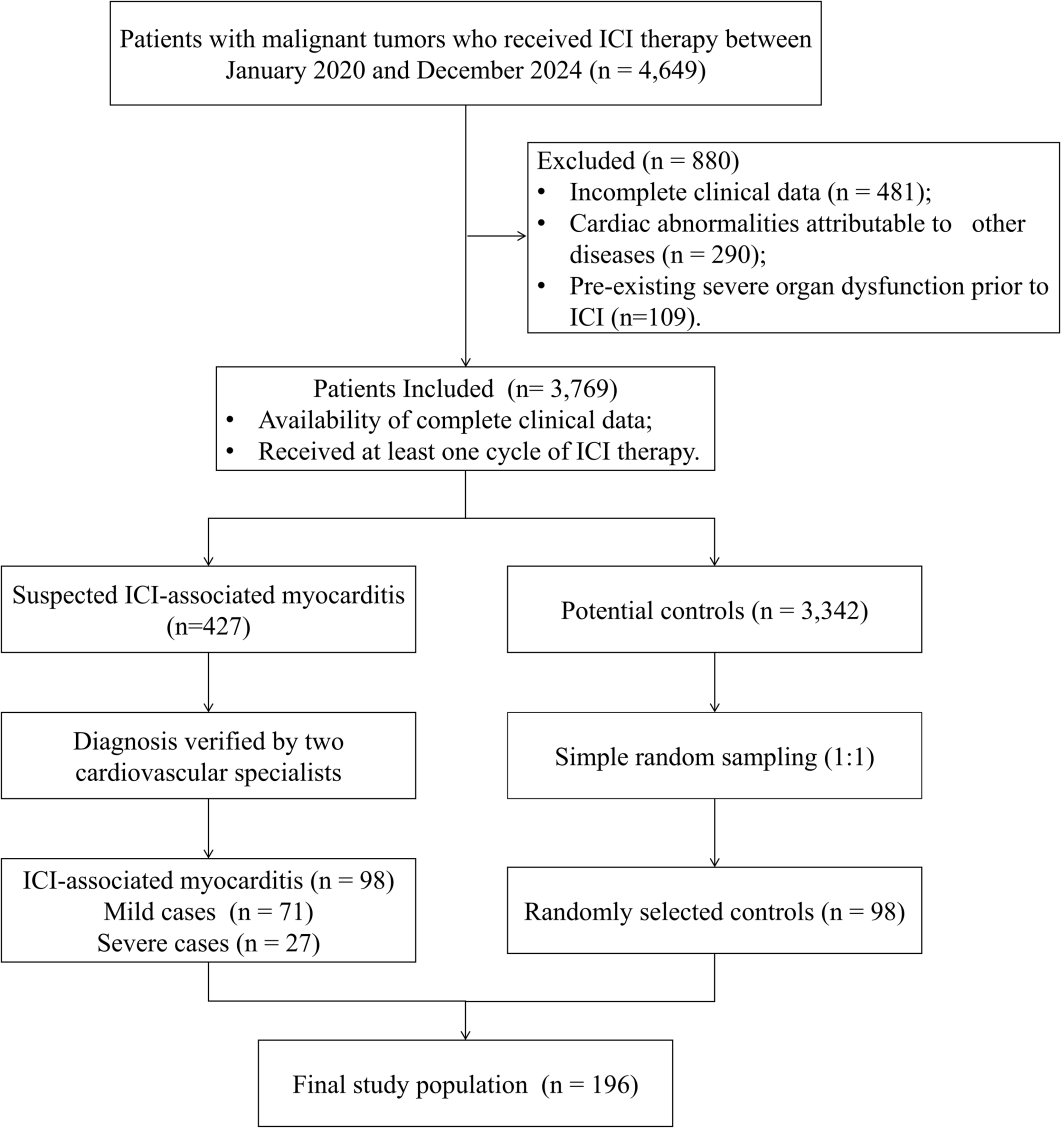
Patient selection flowchart. Flowchart illustrating the selection process of patients with ICI-associated myocarditis and controls. ICI, immune checkpoint inhibitor.

This was a retrospective unmatched case-control study. The study population comprised patients with malignant tumors who received ICI therapy at The Sixth Affiliated Hospital, Sun Yat-sen University, Guangzhou, China between January 2020 and December 2024. The case group include patients diagnosed with ICI-associated myocarditis. Controls were selected from the same patient population using simple random sampling (1:1 ratio) and received ICI therapy during the same period as cases, with confirmed absence of ICI-associated myocarditis. This was an unmatched study. Controls were not pre-selected to match cases on any variables. Regarding confirmation of control status, patients were considered not to have developed ICI-associated myocarditis if they had no clinical suspicion of myocarditis during ICI treatment and follow-up and thus did not undergo diagnostic evaluation. For the those who underwent cardiac biomarker testing or electrocardiography for other clinical reasons, the absence of diagnostic criteria was confirmed based on these results. Inclusion criteria were: [1] availability of complete clinical data; and [2] receipt of at least one cycle of ICI therapy. Exclusion criteria included: [1] incomplete clinical data; [2] abnormal cardiac injury markers or electrocardiogram findings attributable to other diseases; or [3] pre-existing severe organ dysfunction prior to ICI initiation. The sample size was not predetermined by a formal power calculation. As this was a retrospective case-control study, all consecutive patients diagnosed with ICI-associated myocarditis during the study period who met the eligibility criteria were included as cases. The patient selection process is illustrated in a STROBE-compliant flowchart (**Figure 1**). The Ethics Committee of the Sixth Affiliated Hospital, Sun Yat-sen University, Guangzhou, China, approved the study protocol (Approval No. 2024ZSLYEC-466) and waived the requirement for written informed consent.

### 2.2 Data collection

Data for this study were retrospectively extracted from the electronic medical record systems of the participating hospitals. The collected covariates encompassed the following categories: [1] patient demographics and medical history; [2] cancer treatment-related information: cancer type, type of ICI administered, combination medications, and line of therapy; [3] cardiac-related parameters: cardiac enzyme profiles and ECG results; and [4] other laboratory parameters, including complete blood count, hepatic and renal function panels, blood glucose, urinary protein, and coagulation profiles. For patients in the myocarditis group, data were collected at two distinct time points: before the initiation of any ICI therapy and at the time of myocarditis diagnosis. In the control group, data were collected at a single time point prior to the first ICI treatment.

### 2.3 Definitions and outcomes of interest

According to the 2025 European Society of Cardiology Guidelines (25), the diagnosis of myocarditis is classified as follows: definite myocarditis requires compatible clinical presentation confirmed by cardiac magnetic resonance (CMR) or endomyocardial biopsy (EMB); possible myocarditis is defined by clinical presentation with at least one additional diagnostic criterion when CMR or EMB results are uncertain or unavailable; the diagnosis is considered unlikely if clinical presentation occurs without any additional criteria. Additional diagnostic criteria include ECG abnormalities such as ST-T changes, elevated cardiac troponin, imaging abnormalities such as abnormal strain, wall motion abnormalities, reduced ejection fraction, or CMR evidence of myocardial oedema or late gadolinium enhancement. All diagnoses of myocarditis in the case group were independently verified by two cardiovascular specialists. Based on these criteria, both patients with definite and possible ICI-associated myocarditis were included in the case group. Patients with an unlikely diagnosis were excluded. The severity of ICI-associated myocarditis was adjudicated according to the CTCAE version 5.0 (26) and graded as grade 1 to grade 4. For analytical purposes, grades 1 and 2 were combined as mild myocarditis, and grades 3 and 4 were combined as severe myocarditis.

### 2.4 Statistical analysis

Baseline characteristics are presented as continuous or categorical variables. Continuous data are described using mean ± standard deviation or median with interquartile range (IQR), while categorical variables are summarized as frequencies and percentages. The study population was stratified into two primary cohorts: patients with ICI-associated myocarditis (case) and controls. Within the myocarditis cohort, patients were further subcategorized into mild and severe subgroups. For intergroup comparisons, continuous variables were analyzed using Student’s t-test (for normally distributed data) or Mann-Whitney U test (for non-normally distributed data). Categorical variables were compared using the Pearson Chi-square test when all expected cell counts were ≥ 5; Fisher’s exact test was applied when expected cell count < 5. Odds ratios (OR) with 95% confidence intervals (CI) were calculated for all binary comparisons. Variables with *P* < 0.05 in the univariate analysis were entered into a multivariate logistic regression model using forward stepwise (conditional) selection to identify independent predictors. The predictive performance of predictors was assessed by the area under the curve (AUC) derived from receiver operating characteristic (ROC) analysis. To assess internal validity and potential overfitting, we performed bootstrap resampling (1000 replicates) with bias-corrected and accelerated (BCa) confidence intervals for the AUC of the final multivariable model. All statistical analyses were performed using SPSS version 26.0 (IBM Corp., Armonk, NY, USA), with a two-tailed *P*-value < 0.05 considered statistically significant.

## 3 Results

### 3.1 Patient characteristics

A total of 4,649 patients who received ICI therapy at The Sixth Affiliated Hospital, Sun Yat-sen University between January 2020 and December 2024 were initially screened. After excluding ineligible cases according to the flow diagram (**Figure 1**), 98 patients with ICI-associated myocarditis and 98 potential controls were identified, resulting in a final study population of 196 patients. The baseline clinical characteristics are summarized in **Table 1**. Baseline characteristics were well-balanced between the two groups, with no statistically significant differences observed in age, sex, cancer type, specific ICI agent, line of therapy, combination regimens, or cardiovascular risk factors (all *P* > 0.05, **Table 1**). The mean age was 57.83 ± 13.05 years in the myocarditis group and 55.30 ± 12.67 years in the control group. Male patients accounted for 66.33% and 67.35% of the myocarditis and control groups, respectively. The most common cancer types among the enrolled patients were colon cancer, gastric cancer, and esophageal cancer. The majority of patients received first-line therapy. The distribution of ICI types was comparable between the two groups, with sintilimab being the most commonly administered agent in both the myocarditis (32.65%) and control (43.88%) cohorts. The majority of patients in both groups received ICIs in combination with other anticancer therapies, primarily chemotherapy, with no significant difference observed in combined therapy (all *P* > 0.05, **Table 1**). Furthermore, the prevalence of cardiovascular risk factors, including hypertension, diabetes, and prior cardiovascular diseases, was not significantly different between patients who developed myocarditis and those who did not (all *P* > 0.05, **Table 1**).

**Table 1:** Baseline demographic and clinical characteristics of the ICI-associated myocarditis and control groups.

Variables	Total (n = 196)	Myocarditis (n = 98)	Control (n = 98)	OR (95% CI)	*P* value
Age at start of ICI (years), mean ± SD	56.56 ± 12.89	57.83 ± 13.05	55.30 ± 12.67	–	0.170^†^
Male, *n* (%)	131 (66.84)	65 (66.33)	66 (67.35)	1.05 (0.58-1.90)	0.879^ǂ^
Weight (Kg), mean ± SD	59.74 ± 11.24	60.63 ± 10.23	58.84 ± 12.16	–	0.268^†^
Types of cancer, n (%)					
Colon cancer	38 (19.39)	17 (17.35)	21 (21.43)	0.77 (0.38-1.57)	0.470^ǂ^
Gastric cancer	38 (19.39)	17 (17.35)	21 (21.43)	0.77 (0.38-1.57)	0.470^ǂ^
Esophageal cancer	36 (18.37)	16 (16.33)	20 (20.41)	0.76 (0.37-1.58)	0.461^ǂ^
Rectal cancer	31 (15.82)	13 (13.27)	18 (18.37)	0.68 (0.31-1.48)	0.328^ǂ^
Lung cancer	10 (5.10)	7 (7.14)	3 (3.06)	2.44 (0.61-9.71)	0.194^ǂ^
Liver cancer	6 (3.06)	4 (4.08)	2 (2.04)	2.04 (0.37-11.42)	0.683^§^
Melanoma	4 (2.04)	3 (3.06)	1 (1.02)	3.06 (0.31-29.97)	0.621^§^
Others	33 (16.84)	21 (21.43)	12 (12.24)	1.96 (0.90-4.23)	0.086^ǂ^
Treatment lines of ICI, *n* (%)					
Neoadjuvant therapy	52 (26.53)	28 (28.57)	24 (24.49)	1.23 (0.65-2.33)	0.518^ǂ^
Adjuvant therapy	14 (7.14)	5(5.10)	9 (9.18)	0.53 (0.17-1.65)	0.267^ǂ^
First-line therapy	95 (48.47)	45 (45.92)	50 (51.02)	0.82 (0.47-1.43)	0.475^ǂ^
Second-line therapy	18 (9.18)	10 (10.20)	8 (8.16)	1.28 (0.48-3.39)	0.621^ǂ^
≥Third-line therapy	17 (8.67)	10 (10.20)	7 (7.14)	1.48 (0.54-4.05)	0.446^ǂ^
Type of ICIs, *n* (%)					
Sintilimab	75 (38.27)	32 (32.65)	43 (43.88)	0.62 (0.35-1.11)	0.106^ǂ^
Toripalimab	28 (14.29)	17 (17.35)	11 (11.22)	1.66 (0.73-3.76)	0.221^ǂ^
Tislelizumab	28 (14.29)	17 (17.35)	11 (11.22)	1.66 (0.73-3.76)	0.221^ǂ^
Camrelizumab	26 (13.27)	9 (9.18)	17 (17.35)	0.48 (0.20-1.14)	0.092^ǂ^
Others	39 (19.90)	23 (23.47)	16 (16.33)	1.57 (0.77-3.20)	0.210^ǂ^
Combined therapy, n (%)					
ICI monotherapy	26 (13.27)	14 (14.29)	12 (12.24)	1.19 (0.52-2.73)	0.674^ǂ^
Chemotherapy	115 (58.67)	60 (61.22)	55 (56.13)	1.23 (0.70-2.18)	0.468^ǂ^
Targeted-therapy	17 (8.67)	9 (9.18)	8 (8.16)	1.14 (0.42-3.08)	0.800^ǂ^
Chemotherapy plus targeted therapy	38 (19.39)	15 (15.31)	23 (23.47)	0.59 (0.29-1.21)	0.148^ǂ^
Cardiovascular risk factors, *n* (%)					
Hypertension	41 (20.92)	26 (26.53)	15 (15.31)	2.00 (0.98-4.06)	0.053^ǂ^
Diabetes	13 (6.63)	6 (6.12)	7 (7.14)	0.85 (0.27-2.62)	0.774^ǂ^
Prior CV diseases	22 (11.22)	13 (13.27)	9 (9.18)	1.51 (0.62-3.72)	0.365^ǂ^

Values are mean ± SD, n (%).

† Student’s t-test; ǂ Pearson Chi-square test; § Fisher’s exact test. For categorical variables, Pearson Chi-square test was used when all expected cell counts ≥ 5; Fisher’s exact test was applied when expected cell count < 5.

ICI, immune checkpoint inhibitor; OR, odds ratio; CI, confidence interval; SD, standard deviation; CV, cardiovascular.

Within the myocarditis cohort, patients were classified into the mild myocarditis subgroup (n = 71, 72.45%) and the severe myocarditis subgroup (n = 27, 27.55%). Their baseline clinical characteristics are presented in **Supplementary Table 1**. The demographic and clinical features were well-balanced between the mild and severe myocarditis subgroups at baseline, with no statistically significant differences observed (all *P* > 0.05, **Supplementary Table 1**).

### 3.2 Baseline risk prediction model for ICI-associated myocarditis

**Figure 2:**
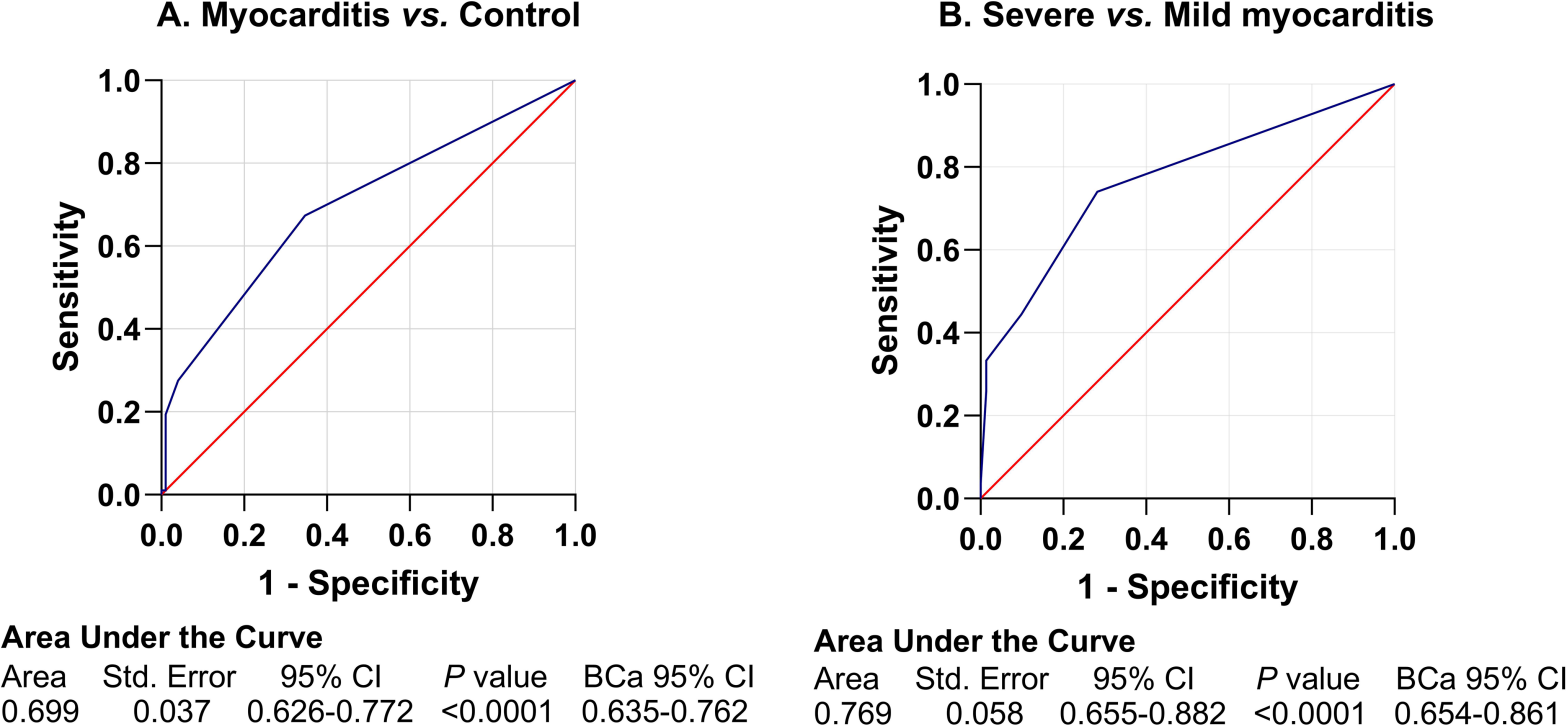
Predictive performance of logistic regression models for ICI-associated myocarditis. **(A)** ROC curve for predicting ICI-associated myocarditis (cases *vs.* controls). The AUC was 0.699 (model-based 95% CI: 0.626–0.772; BCa 95% CI: 0.635–0.762). **(B)** ROC curve for predicting severe myocarditis (severe *vs.* mild cases). The AUC was 0.769 (model-based 95% CI: 0.655–0.882; BCa 95% CI: 0.654–0.861). Model-based CIs were calculated based on the standard errors from the logistic regression model; BCa CIs were calculated using 1,000 bootstrap resamples. ICI, immune checkpoint inhibitor; ROC, receiver operating characteristic; AUC, area under the curve; CI, confidence interval; BCa, bias-corrected and accelerated.

Comparative analysis of pre-ICI baseline characteristics revealed significant differences between the myocarditis cohort and controls, as detailed in **Table 2**. Compared with the control group, the myocarditis group had a significantly higher proportion of patients with a lymphocyte percentage below the lower limit of normal (LLN, 55.10% *vs.* 30.61%, OR = 2.78, 95% CI: 1.55-5.00, *χ*² (1 d.f.) = 12.00, *P* = 0.001) and a higher proportion with an eosinophil ratio above the upper limit of normal (ULN, 18.37% *vs.* 4.08%, OR = 5.29, 95% CI: 1.72-16.27, *χ*² (1 d.f.) = 10.04, *P* = 0.002). Conversely, a lower proportion of myocarditis patients exhibited an elevated monocyte ratio (14.29% *vs.* 27.55%, OR = 0.44, 95% CI: 0.21-0.90, *χ*² (1 d.f.) = 5.21, *P* = 0.022). Furthermore, the myocarditis group showed a higher incidence of elevated myoglobin (MYO) levels > ULN (10.20% *vs.* 1.02%, OR = 11.02, 95% CI: 1.38-87.86, *χ*² (1 d.f.) = 7.80, *P* = 0.005). Multivariable analysis identified that a low lymphocyte ratio, a high eosinophil ratio, and elevated myoglobin as factors associated with the occurrence of ICI-associated myocarditis. The ROC curve was used to evaluate the predictive value of the model, with an AUC of 0.699 (95% CI, 0.626-0.772, *P* < 0.0001 for the test of AUC > 0.5, **Figure 2A**). Bootstrap internal validation with 1000 replicates yielded a BCa 95% CI of 0.635-0.762 for the AUC.

**Table 2:** Baseline predictors of ICI-associated myocarditis in multivariable analysis.

Variables	Myocarditis (n = 98)	Control (n = 98)	Univariate analyses		Multivariable analysis	
			OR (95% CI)	*P*	OR (95% CI)	*P*
Laboratory hematologic parameters, n (%)						
Lymphocyte ratio < LLN	54 (55.10)	30 (30.61)	2.78 (1.55-5.00)	0.001^ǂ^	2.87 (1.55-5.30)	0.001^¶^
Monocyte ratio > ULN	14 (14.29)	27 (27.55)	0.44 (0.21-0.90)	0.022^ǂ^	–	0.090^¶^
Eosinophil ratio > ULN	18 (18.37)	4 (4.08)	5.29 (1.72-16.27)	0.002^ǂ^	5.29 (1.72-16.27)	0.004^¶^
Laboratory cardiac biomarkers, n (%)						
MYO > ULN	10 (10.20)	1 (1.02)	11.02 (1.38-87.86)	0.005^ǂ^	11.02 (1.38-87.86)	0.029^¶^

Values are n (%).

ǂ Pearson Chi-square test; ¶ Logistic regression. Multivariable analysis adjusted for: lymphocyte ratio < LLN, monocyte ratio > ULN, eosinophil ratio > ULN, and MYO > ULN (all variables with *P* < 0.05 in univariate analysis). Forward stepwise conditional logistic regression was used for variable selection.

ICI, immune checkpoint inhibitor; OR, odds ratio; CI, confidence interval; LLN, lower limit of normal; ULN, upper limit of normal; MYO, myoglobin.

### 3.3 Characteristics of mild and severe myocarditis

#### 3.3.1 Onset timing and clinical presentation

**Figure 3:**
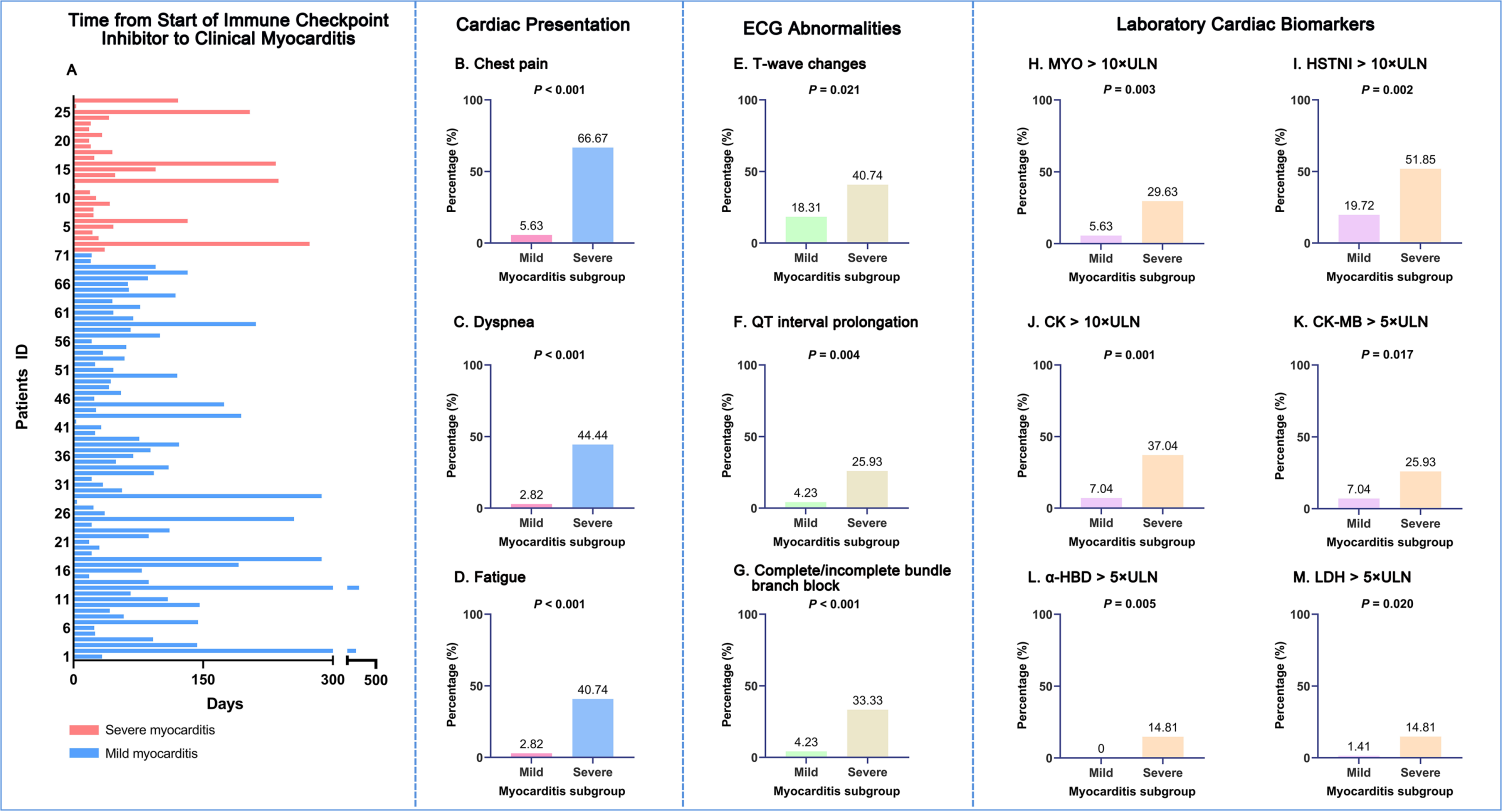
Comparative clinical profiles of mild versus severe ICI-associated myocarditis. **(A)** Swimmer’s plot showing time from ICI initiation to myocarditis onset. Each horizontal bar represents one patient. The median time to onset was significantly shorter in the severe myocarditis group compared to the mild group (33 days *vs.* 63 days, Mann-Whitney *U* = 703.50, *P* = 0.043). **(B–D)** Prevalence of key cardiac symptoms at presentation: chest pain **(B)**, dyspnea **(C)**, and fatigue **(D–G)** ECG parameters: T-wave changes **(E)**, QT interval prolongation **(F)**, and complete/incomplete bundle branch block **(G)**. **(H–M)** Extreme elevations in laboratory biomarkers: MYO> 10 × ULN **(H)**, HSTNI> 10 × ULN **(I)**, CK>10 × ULN **(J)**, CK-MB> 5 × ULN **(K)**, α-HBD> 5 × ULN **(L)**, and LDH >5 × ULN **(M)**. For panels B-M, data are presented as percentages; *P*-values were calculated using the Pearson Chi-square test or Fisher’s exact test, as appropriate. ICI, immune checkpoint inhibitor; ECG, electrocardiogram; MYO, myoglobin; ULN, upper limit of normal; HSTNI, high-sensitivity troponin I; CK, creatine kinase; CK-MB, creatine kinase-myocardial band; α-HBD, alpha-hydroxybutyrate dehydrogenase; LDH, lactate dehydrogenase.

Characteristics of mild and severe myocarditis subgroups are presented in **Supplementary Table 2**. The median time to myocarditis onset across all patients was 48.5 days. **Figure 3A** shows the time to onset of myocarditis from the initiation of ICI therapy in the myocarditis group. Patients with severe myocarditis experienced a significantly earlier onset compared to those with the mild form (33 days [IQR 75] *vs.* 63 days [IQR 80], Mann-Whitney *U* = 703.50, *P* = 0.043). The severe myocarditis subgroup demonstrated a significantly higher frequency of typical cardiac symptoms than the mild subgroup, including chest pain (66.67% *vs.* 5.63%, OR = 33.50, 95% CI: 9.24-121.41, *χ*² (1 d.f.) = 41.85, *P* < 0.001, **Figure 3B**), dyspnea (44.44% *vs.* 2.82%, Fisher’s exact test, *P* < 0.001, **Figure 3C**), and fatigue (40.74% *vs.* 2.82%, Fisher’s exact test, *P* < 0.001, **Figure 3D**).

#### 3.3.2 ECG and cardiac biomarkers

Abnormal ECG findings were present in 57.14% (56/98) of myocarditis patients. Detailed ECG abnormalities are presented in **Supplementary Table 2**. Specifically, the severe subgroup exhibited a higher prevalence of T-wave changes (40.74% *vs.* 18.31%, OR = 3.07, 95% CI: 1.16-8.13, *χ*² (1 d.f.) = 5.32, *P* = 0.021, **Figure 3E**), QT interval prolongation (25.93% *vs.* 4.23%, Fisher’s exact test, *P* = 0.004, **Figure 3F**), and complete/incomplete bundle branch block (33.33% *vs.* 4.23%, Fisher’s exact test, *P* < 0.001, **Figure 3G**). Echocardiography was abnormal in 13.27% (13/98) of all the myocarditis patients, though no significant difference in left ventricular ejection fraction (LVEF) was observed between the severe and mild subgroups (66% [IQR 16.50] *vs.* 67% [IQR 7.25], Mann-Whitney *U* = 259.50, *P* = 0.287).

Cardiac biomarkers at myocarditis diagnosis were markedly higher in the severe subgroup than in the mild subgroup. As presented in **Supplementary Table 2**, a significantly higher proportion of patients with severe disease exhibited extreme elevations in key biomarkers. Specifically, the severe subgroup had a greater prevalence of: MYO >10 × ULN (29.63% *vs.* 5.63%, Fisher’s exact test, *P* = 0.003, **Figure 3H**), high-sensitivity troponin I (HSTNI) >10 × ULN (51.85% *vs.* 19.72%, OR = 4.39, 95% CI: 1.69-11.39, *χ*² (1 d.f.) = 9.90, *P* = 0.002, **Figure 3I**), creatine kinase (CK) >10 × ULN (37.04% *vs.* 7.04%, Fisher’s exact test, *P* = 0.001, **Figure 3J**), CK-MB >5 × ULN (25.93% *vs.* 7.04%, Fisher’s exact test, *P* = 0.017, **Figure 3K**), α-Hydroxybutyrate dehydrogenase (α-HBD) >5 × ULN (14.81% *vs.* 0%, Fisher’s exact test, *P* = 0.005, **Figure 3L**), and lactate dehydrogenase (LDH) > 5× ULN (14.81% *vs.* 1.41%, Fisher’s exact test, *P* = 0.020, **Figure 3M**).

#### 3.3.3 Concurrent irAEs

The incidence of concurrent irAEs, such as myositis (22.22% *vs.* 1.41%, Fisher’s exact test, *P* = 0.002), pneumonia (25.93% *vs.* 0%, Fisher’s exact test, *P* < 0.001) and hepatitis (55.56% *vs.* 30.99%, OR = 2.78, 95% CI: 1.12-6.92, *χ*² (1 d.f.) = 5.02, *P* = 0.025), was significantly higher in patients with severe myocarditis, presented in **Supplementary Table 2**.

#### 3.3.4 Predictive model for severe ICI-associated myocarditis

As presented in **Table 3**, the logistic regression analysis identified three key factors that were significantly associated with severe myocarditis and incorporated into the final predictive model: T-wave changes, complete/incomplete bundle branch block, and a CK level >10× ULN. The ROC curve was used to evaluate the predictive value of the model, with an AUC of 0.769 (95% CI, 0.655-0.882, *P* < 0.0001 for the test of AUC > 0.5, **Figure 2B**). Bootstrap internal validation with 1000 replicates yielded a BCa 95% CI of 0.654-0.861 for the AUC, indicating acceptable model stability despite the limited sample size.

**Table 3:** Predictors at onset for severe ICI-associated myocarditis in multivariable analysis.

Variables	Mild myocarditis (n = 71)	Severe myocarditis (n = 27)	Univariate analyses		Multivariable analysis	
			OR (95% CI)	*P*	OR (95% CI)	*P*
Time from ICI initiation to onset of myocarditis (days), median (IQR)	63 (80)	33 (75)	–	0.043^£^	–	0.549^¶^
New onset ECG abnormalities, *n* (%)						
T-wave changes	13 (18.31)	11 (40.74)	3.07 (1.16-8.13)	0.021^ǂ^	5.44 (1.75-16.91)	0.003^¶^
QT interval prolongation	3 (4.23)	7 (25.93)	7.93 (1.87-33.53)	0.004^§^	–	0.169^¶^
Complete/incomplete bundle branch block	3 (4.23)	9 (33.33)	11.33 (2.78-46.24)	< 0.001^§^	7.12 (1.37-36.92)	0.019^¶^
Laboratory cardiac biomarkers, *n* (%)						
MYO, ng/mL						
> 10 × ULN	4 (5.63)	8 (29.63)	7.05 (1.92-25.98)	0.003^§^	–	0.617^¶^
HSTNI, ng/mL						
> 10 × ULN	14 (19.72)	14 (51.85)	4.39 (1.69-11.39)	0.002^ǂ^	–	0.193^¶^
CK, U/L						
> 10 × ULN	5 (7.04)	10 (37.04)	7.77 (2.34-25.74)	0.001^§^	5.57 (1.28-24.26)	0.022^¶^
CK-MB, U/L						
> 5 × ULN	5 (7.04)	7 (25.93)	4.62 (1.32-16.16)	0.017^§^	–	0.239^¶^
α-HBD, U/L						
> 5 × ULN	0 (0)	4 (14.81)	–	0.005^§^	–	0.129^¶^
LDH, U/L						
> 5 × ULN	1 (1.41)	4 (14.81)	12.17 (1.29-114.51)	0.020^§^	–	0.099^¶^

Values are median (IQR) or n (%).

£ Mann-Whitney U test; ǂ Pearson Chi-square test; § Fisher’s exact test; ¶ Logistic regression. For categorical variables, Pearson Chi-square test was used when all expected cell counts ≥ 5; Fisher’s exact test was applied when expected cell count < 5. Multivariable analysis adjusted for: time from ICI initiation to onset, T-wave changes, QT interval prolongation, bundle branch block, MYO> 10 × ULN, HSTNI> 10 × ULN, CK> 10 × ULN, CK-MB> 5 × ULN, α-HBD> 5 × ULN, and LDH > 5 × ULN (all variables with *P* < 0.05 in univariate analysis). Forward stepwise conditional logistic regression was used for variable selection.

ICI, immune checkpoint inhibitor; OR, odds ratio; CI, confidence interval; IQR, interquartile range; ECG, electrocardiogram; MYO, myoglobin; HSTNI, high-sensitivity troponin I; CK, creatine kinase; CK-MB, creatine kinase MB; α-HBD, α-hydroxybutyrate dehydrogenase; LDH, lactate dehydrogenase.

## 4 Discussion

The clinical management of ICI-associated myocarditis remains challenging despite growing evidence on risk factors and prognostic indicators. Previous studies (13–19) have reported associations between outcomes and cardiac biomarkers, ECG abnormalities, and the use of dual ICI therapy. Building upon this existing evidence, this real-world, retrospective case-control study aimed to further explore risk stratification. The findings indicate that the baseline elevated eosinophil ratio, reduced lymphocyte ratio, and elevated myoglobin collectively may constitute risk factors for ICI-associated myocarditis. Furthermore, at clinical presentation, a combination of specific ECG abnormalities and marked CK elevation (>10 × ULN) was associated with progression to severe myocarditis. The baseline model for predicting ICI-associated myocarditis showed moderate discriminative ability (AUC 0.699), while the model for predicting severe myocarditis showed improved performance (AUC 0.769). These findings represent an initial step toward risk stratification and require external validation before clinical application.

This study suggests that an elevated baseline eosinophil ratio, reduced lymphocyte ratio, and elevated myoglobin may collectively form a predictive profile for ICI-associated myocarditis. This integrated model is consistent with a predisposing background characterized by both a dysregulated immune system and a vulnerable target organ. Regarding the eosinophil elevation, this may reflect a pre-existing Th2-polarized immune state (27). Such an immune microenvironment, when systemically activated by ICIs, could lower the threshold for breaking self-tolerance in specific tissues like the heart (28). Furthermore, eosinophils are capable of releasing a repertoire of pre-formed cytokines, chemokines, and granule proteins, such as major basic protein, eosinophil cationic protein (29), which contribute to the initiation and amplification of cardiac inflammation through direct cytotoxic effects and recruitment of other immune cells (30). The reduced lymphocyte ratio in the model may reflect a baseline state of immunosuppression or exhaustion, impairing the regulation of aberrant immune responses (31); it is also consistent with the migration of activated, cardiac-specific T-cells from the circulation to infiltrate the myocardial tissue, a mechanism aligned with previous reports linking baseline lymphopenia to ICI myocarditis (18). Additionally, elevated baseline myoglobin suggests potential subclinical injury to cardiac or skeletal muscle (32, 33). These pre-existing micro-injuries may enhance the inflammatory response during ICI-triggered immune attack. Regarding eosinophils and disease severity, we observed no significant correlation between eosinophil ratio and myocarditis severity in this cohort. This may reflect limited statistical power due to the small severe subgroup (n=27) or a greater role for eosinophils in initiation rather than amplification of inflammation. Collectively, this multi-parameter model suggests an additional perspective on the pathogenesis of ICI myocarditis that extends beyond the conventional T-cell-centric view (34, 35). However, this study did not account for other potential confounders, such as pre-existing inflammatory conditions or corticosteroid use, which may influence baseline hematological parameters. Prospective studies with comprehensive disease and medication histories are needed to confirm these findings.

These data further described the clinical features associated with different severities of ICI-associated myocarditis. The proportion of severe myocarditis in our cohort (27.6%, 27/98) is consistent with previously reported rates (14, 36), suggesting minimal sampling bias. Notably, patients with severe myocarditis exhibited a significantly earlier onset compared to those with mild cases (33 *vs.* 63 days), which aligns with the fulminant myocarditis phenotype described by previous research (9, 37) and may reflect a more intense immune activation mechanism. However, cross-cohort comparisons are limited by variations in diagnostic criteria and ICI regimens.

The present analysis found that the co-occurrence of specific ECG abnormalities and extreme CK elevation (>10 × ULN) was associated with severe myocarditis, offering a potential tool for risk stratification. Regarding biomarkers, high-sensitivity troponin is widely used for diagnosing myocardial injury due to its cardiac specificity (38). However, the present findings suggest that extreme CK elevation (>10 × ULN) may add value in predicting myocarditis severity. This may indicate that prognostic assessment depends not only on the specificity of myocardial injury but also on its extent. While troponin indicates cardiomyocyte injury (39), extreme CK elevation often suggests more widespread myocyte necrosis. This could be explained by concurrent myositis or an intense inflammatory response within the myocardium itself, leading to extensive CK release (9, 40). Thus, extreme CK elevation may serve as a marker of extensive myocyte necrosis, a feature of severe disease.

Similarly, while existing literature describes various ECG features in myocarditis patients (15, 41), this study observed that T-wave changes and bundle branch blocks were associated with severe ICI-associated myocarditis. T-wave alterations may reflect diffuse abnormalities in myocardial repolarization, suggesting widespread ischemia or inflammatory infiltration (42). Bundle branch blocks may indicate conduction interruption within the His-Purkinje system, potentially resulting from acute inflammatory edema or necrosis (43). Possible mechanisms linking severe ICI-associated myocarditis to these ECG abnormalities include: [1] inflammation-driven fibrosis creating a substrate for conduction delays and block (44); [2] direct interference of inflammatory infiltrates with the cardiac conduction system (45); and [3] cytokine- or autoantibody-induced dysfunction of cardiac ion channels affecting repolarization (46). The association of these ECG abnormalities with severe disease suggests more extensive inflammatory burden and myocardial involvement, and may reflect underlying inflammatory electrophysiological remodeling. Consequently, the proposed composite model of “electrophysiological instability coupled with extensive myocyte necrosis” offers an integrated approach for severity assessment that extends beyond sole reliance on troponin to include electrical and structural consequences of myocardial inflammation.

The higher incidence of concurrent non-cardiac irAEs (e.g., hepatitis, myositis, pneumonia) in patients with severe myocarditis supports the hypothesis of a systemic hyper-activated immune state. This finding is consistent with the baseline immune dysregulation suggested by an elevated eosinophil ratio and the more intense inflammatory response observed at myocarditis onset. However, conventional diagnostic tests have limited specificity in irAEs. Severe myocarditis itself can elevate CK and transaminases, which may lead to false diagnoses of concurrent myositis or hepatitis. Thus, apparent multi-organ involvement in ICI-treated patients may represent severe myocarditis with associated myositis rather than true multi-organ irAEs. Future studies should employ more specific modalities, such as tissue biopsy or novel biomarkers, to accurately distinguish these entities.

This study proposes a two-tiered risk-prediction models, but has several limitations. First, inherent biases exist in the retrospective case-control design. Selection bias may arise from both the unmatched design and the restriction of controls to patients with available cardiac biomarkers and CK levels, tests not routinely performed in immunotherapy recipients, potentially compromising the representativeness of the control group. Observation bias could arise from differential clinical monitoring, potentially leading to more frequent event detection in cases and overestimation of associations. Information bias may result from the lack of follow-up data in controls (single time-point) compared to cases (two time-point), limiting the comparability of time-varying covariates. Thus, these findings should be considered hypothesis-generating and require prospective validation. Second, exclusion of patients with severe organ dysfunction prior to ICI initiation limits generalizability, as findings may not apply to higher-risk populations with comorbidities. The modest sample size, particularly the severe myocarditis subgroup (n=27), limited statistical power, increasing type II error risk. However, key predictors showed relatively large effect sizes and remained significant. *Post-hoc* power calculations were not performed due to methodological concerns. Third, this single-center Chinese cohort, treated predominantly with sintilimab, toripalimab, and tislelizumab rather than Western regimens, limits direct comparability, highlighting the need for multi-center external validation. Finally, the precise mechanistic roles of eosinophils and lymphocytes in ICI-associated myocarditis pathogenesis and their interactions with other immune cells remain to be elucidated by further basic research.

## 5 Conclusion

In conclusion, this study describes an integrated risk stratification strategy for ICI-associated myocarditis. A baseline hematological profile characterized by elevated eosinophil ratio, reduced lymphocyte ratio, and elevated myoglobin may help identify at-risk patients prior to ICI therapy, while a combination of specific ECG abnormalities and extreme CK elevation at presentation may predict severe myocarditis. These findings support a two-stage approach to early risk stratification and targeted management.

## Data Availability

The original contributions presented in the study are included in the article/**Supplementary Material**. Further inquiries can be directed to the corresponding author.

## References

[1] Pang K Shi ZD Wei LY Dong Y Ma YY Wang W . Research progress of therapeutic effects and drug resistance of immunotherapy based on PD-1/PD-l1 blockade. Drug Resist Update. (2023) 66:100907. doi: 10.1016/j.drup.2022.100907. PMID: 36527888

[2] Berner F Bomze D Diem S Ali OH Fassler M Ring S . Association of checkpoint inhibitor-induced toxic effects with shared cancer and tissue antigens in non-small cell lung cancer. JAMA Oncol. (2019) 5:1043–7. doi: 10.1001/jamaoncol.2019.0402. PMID: 31021392 PMC6487908

[3] Michot JM Bigenwald C Champiat S Collins M Carbonnel F Postel-Vinay S . Immune-related adverse events with immune checkpoint blockade: a comprehensive review. Eur J Cancer. (2016) 54:139–48. doi: 10.1016/j.ejca.2015.11.016. PMID: 26765102

[4] Zhou X Yao Z Bai H Duan J Wang Z Wang X . Treatment-related adverse events of PD-1 and PD-l1 inhibitor-based combination therapies in clinical trials: a systematic review and meta-analysis. Lancet Oncol. (2021) 22:1265–74. doi: 10.1016/S1470-2045(21)00333-8. PMID: 34391508

[5] Rajha E Chaftari P Kamal M Maamari J Chaftari C Yeung SJ . Gastrointestinal adverse events associated with immune checkpoint inhibitor therapy. Gastroenterol Rep (Oxf). (2019) 8:25–30. doi: 10.1093/gastro/goz065. PMID: 32104583 PMC7034236

[6] Postow MA Sidlow R Hellmann MD . Immune-related adverse events associated with immune checkpoint blockade. N Engl J Med. (2018) 378:158–68. doi: 10.1056/NEJMra1703481. PMID: 29320654

[7] Pi JK Chen XT Zhang YJ Chen XM Wang YC Xu JY . Insight of immune checkpoint inhibitor related myocarditis. Int Immunopharmacol. (2024) 143:113559. doi: 10.1016/j.intimp.2024.113559. PMID: 39536487

[8] Rubio-Infante N Ramirez-Flores YA Castillo EC Lozano O Garcia-Rivas G Torre-Amione G . A systematic review of the mechanisms involved in immune checkpoint inhibitors cardiotoxicity and challenges to improve clinical safety. Front Cell Dev Biol. (2022) 10:851032. doi: 10.3389/fcell.2022.851032. PMID: 35433707 PMC9006991

[9] Salem JE Manouchehri A Moey M Lebrun-Vignes B Bastarache L Pariente A . Cardiovascular toxicities associated with immune checkpoint inhibitors: an observational, retrospective, pharmacovigilance study. Lancet Oncol. (2018) 19:1579–89. doi: 10.1016/S1470-2045(18)30608-9. PMID: 30442497 PMC6287923

[10] Patel RP Parikh R Gunturu KS Tariq RZ Dani SS Ganatra S . Cardiotoxicity of immune checkpoint inhibitors. Curr Oncol Rep. (2021) 23:79. doi: 10.1007/s11912-021-01070-6. PMID: 33937956 PMC8088903

[11] Wang DY Salem JE Cohen JV Chandra S Menzer C Ye F . Fatal toxic effects associated with immune checkpoint inhibitors: a systematic review and meta-analysis. JAMA Oncol. (2018) 4:1721–8. doi: 10.1001/jamaoncol.2018.3923. PMID: 30242316 PMC6440712

[12] Moslehi JJ Salem JE Sosman JA Lebrun-Vignes B Johnson DB . Increased reporting of fatal immune checkpoint inhibitor-associated myocarditis. Lancet. (2018) 391:933. doi: 10.1016/S0140-6736(18)30533-6. PMID: 29536852 PMC6668330

[13] Power JR Dolladille C Ozbay B Procureur A Ederhy S Palaskas NL . Immune checkpoint inhibitor-associated myocarditis: a novel risk score. Eur Heart J. (2025) 47:1050–62. doi: 10.1093/eurheartj/ehaf315. PMID: 40569849 PMC13291914

[14] Xu L Xu M Sun W Zhang W Song Z . Clinical characteristics and prognostic impact of immune checkpoint inhibitor-associated myocarditis in advanced non-small cell lung cancer. Invest New Drugs. (2023) 41:816–24. doi: 10.1007/s10637-023-01400-4. PMID: 37902905

[15] Zlotoff DA Hassan M Zafar A Alvi RM Awadalla M Mahmood SS . Electrocardiographic features of immune checkpoint inhibitor associated myocarditis. J Immunother Cancer. (2021) 9:e002007. doi: 10.1136/jitc-2020-002007. PMID: 33653803 PMC7929895

[16] Xu Y Song Y Liu X Shi Y Liu Y Qian H . Prediction of major adverse cardiac events is the first critical task in the management of immune checkpoint inhibitor-associated myocarditis. Cancer Commun (Lond). (2022) 42:902–5. doi: 10.1002/cac2.12320. PMID: 35678260 PMC9456696

[17] O'Shea MP Karikalan SA Yusuf A Barry T Habib E O'Shea J . Complete heart block is a significant predictor of mortality in immune checkpoint inhibitor myocarditis. Cardiooncology. (2023) 9:34. doi: 10.1186/s40959-023-00185-y. PMID: 37730763 PMC10510176

[18] Drobni ZD Zafar A Zubiri L Zlotoff DA Alvi RM Lee C . Decreased absolute lymphocyte count and increased neutrophil/lymphocyte ratio with immune checkpoint inhibitor-associated myocarditis. J Am Heart Assoc. (2020) 9:e018306. doi: 10.1161/JAHA.120.018306. PMID: 33190570 PMC7763791

[19] Zhuang Y An Q Wang F Han D Qiao Z Jiang Q . The role of circulating biomarkers in predicting the 30-day mortality of immune checkpoint inhibitors-related myocarditis: a retrospective cohort study. Intern Emerg Med. (2024) 19:377–89. doi: 10.1007/s11739-023-03481-8. PMID: 38085435

[20] Lindsay AC Walker AM Schneeweiss S . Myocarditis in patients starting combination checkpoint inhibitor therapy: analysis of a commercial claims database. J Am Heart Assoc. (2025) 14:e035689. doi: 10.1161/JAHA.124.035689. PMID: 39719418 PMC12054407

[21] Kachi S Shirakashi M Nomizo T Onishi M Toda Kato E Nobashi TW . Immune checkpoint inhibitor-induced myocarditis and multiple adverse events with pre-existing rheumatoid arthritis: a case report and literature review. Immunol Med. (2025) 48:400–6. doi: 10.1080/25785826.2025.2515688. PMID: 40471689

[22] Haanen J Obeid M Spain L Carbonnel F Wang Y Robert C . Management of toxicities from immunotherapy: ESMO clinical practice guideline for diagnosis, treatment and follow-up. Ann Oncol. (2022) 33:1217–38. doi: 10.1016/j.annonc.2022.10.001. PMID: 36270461

[23] Thompson JA Schneider BJ Brahmer J Zaid MA Achufusi A Armand P . NCCN guidelines insights: management of immunotherapy-related toxicities, version 2.2024. J Natl Compr Canc Netw. (2024) 22:582–92. doi: 10.6004/jnccn.2024.0057. PMID: 39536465

[24] Basch E Reeve BB Mitchell SA Clauser SB Minasian LM Dueck AC . Development of the national cancer institute's patient-reported outcomes version of the common terminology criteria for adverse events (PRO-CTCAE). J Natl Cancer Inst. (2014) 106:dju244. doi: 10.1093/jnci/dju244. PMID: 25265940 PMC4200059

[25] Schulz-Menger J Collini V Gröschel J Adler Y Brucato A Christian V . 2025 ESC guidelines for the management of myocarditis and pericarditis. Eur Heart J. (2025) 46:3952–4041. doi: 10.1093/eurheartj/ehaf192. PMID: 40878297

[26] National Institutes Of Health NCI . Common terminology criteria for adverse events (CTCAE) v 5.0 (2017). Available online at: https://ctep.cancer.gov/protocoldevelopment/electronic_applications/docs/CTCAE_v5_Quick_Reference_5x7.pdf (Accessed March 24, 2026).

[27] Jacobsen EA Lee NA Lee JJ . Re-defining the unique roles for eosinophils in allergic respiratory inflammation. Clin Exp Allergy. (2014) 44:1119–36. doi: 10.1111/cea.12358. PMID: 24961290 PMC4148640

[28] Moslehi J Lichtman AH Sharpe AH Galluzzi L Kitsis RN . Immune checkpoint inhibitor-associated myocarditis: manifestations and mechanisms. J Clin Invest. (2021) 131:e145186. doi: 10.1172/JCI145186. PMID: 33645548 PMC7919710

[29] Davoine F Lacy P . Eosinophil cytokines, chemokines, and growth factors: emerging roles in immunity. Front Immunol. (2014) 5:570. doi: 10.3389/fimmu.2014.00570. PMID: 25426119 PMC4225839

[30] Simon S Utikal J Umansky V . Opposing roles of eosinophils in cancer. Cancer Immunol Immunother. (2019) 68:823–33. doi: 10.1007/s00262-018-2255-4. PMID: 30302498 PMC11028063

[31] Kumar P Saini S Prabhakar BS . Cancer immunotherapy with check point inhibitor can cause autoimmune adverse events due to loss of treg homeostasis. Semin Cancer Biol. (2020) 64:29–35. doi: 10.1016/j.semcancer.2019.01.006. PMID: 30716481

[32] Kottwitz J Bruno KA Berg J Salomon GR Fairweather D Elhassan M . Myoglobin for detection of high-risk patients with acute myocarditis. J Cardiovasc Transl Res. (2020) 13:853–63. doi: 10.1007/s12265-020-09957-8. PMID: 32006209 PMC7541375

[33] Maisel AS Templin K Love M Clopton P . A prospective study of an algorithm using cardiac troponin i and myoglobin as adjuncts in the diagnosis of acute myocardial infarction and intermediate coronary syndromes in a veteran's hospital. Clin Cardiol. (2000) 23:915–20. doi: 10.1002/clc.4960231212. PMID: 11129678 PMC6655059

[34] Johnson DB Balko JM Compton ML Chalkias S Gorham J Xu Y . Fulminant myocarditis with combination immune checkpoint blockade. N Engl J Med. (2016) 375:1749–55. doi: 10.1056/NEJMoa1609214. PMID: 27806233 PMC5247797

[35] Grabie N Lichtman AH Padera R . T cell checkpoint regulators in the heart. Cardiovasc Res. (2019) 115:869–77. doi: 10.1093/cvr/cvz025. PMID: 30721928 PMC6452292

[36] Lei Y Zheng X Huang Q Li X Qiu M Liu M . Intrinsic differences in immune checkpoint inhibitor-induced myocarditis: a retrospective analysis of real world data. Front Pharmacol. (2022) 13:914928. doi: 10.3389/fphar.2022.914928. PMID: 35865949 PMC9294234

[37] Ruste V Goldschmidt V Laparra A Messayke S Danlos FX Romano-Martin P . The determinants of very severe immune-related adverse events associated with immune checkpoint inhibitors: a prospective study of the french REISAMIC registry. Eur J Cancer. (2021) 158:217–24. doi: 10.1016/j.ejca.2021.08.048. PMID: 34627664

[38] Thygesen K Alpert JS Jaffe AS Chaitman BR Bax JJ Morrow DA . Fourth universal definition of myocardial infarction (2018). J Am Coll Cardiol. (2018) 72:2231–64. doi: 10.1016/j.jacc.2018.08.1038. PMID: 30153967

[39] Ammirati E Moslehi JJ . Diagnosis and treatment of acute myocarditis: a review. JAMA. (2023) 329:1098–113. doi: 10.1001/jama.2023.3371. PMID: 37014337

[40] Touat M Maisonobe T Knauss S Ben HSO Hervier B Aure K . Immune checkpoint inhibitor-related myositis and myocarditis in patients with cancer. Neurology. (2018) 91:e985–94. doi: 10.1212/WNL.0000000000006124. PMID: 30089619

[41] Song W Zheng Y Dong M Zhong L Bazoukis G Perone F . Electrocardiographic features of immune checkpoint inhibitor-associated myocarditis. Curr Probl Cardiol. (2023) 48:101478. doi: 10.1016/j.cpcardiol.2022.101478. PMID: 36336121

[42] You T Luo C Zhang K Zhang H . Electrophysiological mechanisms underlying t-wave alternans and their role in arrhythmogenesis. Front Physiol. (2021) 12:614946. doi: 10.3389/fphys.2021.614946. PMID: 33746768 PMC7969788

[43] Tan NY Witt CM Oh JK Cha YM . Left bundle branch block: current and future perspectives. Circ Arrhythm Electrophysiol. (2020) 13:e008239. doi: 10.1161/CIRCEP.119.008239. PMID: 32186936

[44] Suthahar N Meijers WC Sillje H De Boer RA . From inflammation to fibrosis-molecular and cellular mechanisms of myocardial tissue remodelling and perspectives on differential treatment opportunities. Curr Heart Fail Rep. (2017) 14:235–50. doi: 10.1007/s11897-017-0343-y. PMID: 28707261 PMC5527069

[45] Hulsmans M Clauss S Xiao L Aguirre AD King KR Hanley A . Macrophages facilitate electrical conduction in the heart. Cell. (2017) 169:510–22. doi: 10.1016/j.cell.2017.03.050. PMID: 28431249 PMC5474950

[46] Lazzerini PE Capecchi PL Laghi-Pasini F Boutjdir M . Autoimmune channelopathies as a novel mechanism in cardiac arrhythmias. Nat Rev Cardiol. (2017) 14:521–35. doi: 10.1038/nrcardio.2017.61. PMID: 28470179

