# Supplementary material for: Integrated risk stratification for ICI-associated myocarditis: a baseline hematological profile and a combined ECG and enzymatic signature at onset
Source: Front Immunol. 2026 Mar 31;17:1762144. doi: 10.3389/fimmu.2026.1762144 (PMC13076345; doi:10.3389/fimmu.2026.1762144)
Supplement: Supplementary file 1 [file Table1.docx]

Supplementary Table 1. Baseline demographic and clinical characteristics of the mild and severe ICI-associated myocarditis subgroups

| **Variables** | **Mild myocarditis**  **(n = 71)** | **Severe myocarditis**  **(n = 27)** | **OR (95% CI)** | ***P* value** |
| --- | --- | --- | --- | --- |
| **Age at start of ICI (years) , mean ± SD** | 58.14 ± 12.63 | 57.00 ± 14.32 | - | 0.701^†^ |
| **Male, *n* (%)** | 47 (66.20) | 18 (66.67) | 0.98 (0.38-2.51) | 0.965^ǂ^ |
| **Weight (Kg) , mean ± SD** | 61.36 ± 10.19 | 58.74 ± 10.27 | - | 0.259^†^ |
| **Types of cancer, *n* (%)** | | | | |
| Colon cancer | 9 (12.68) | 8 (29.63) | 2.90 (0.98-8.56) | 0.071^§^ |
| Gastric cancer | 9 (12.68) | 8 (29.63) | 2.90 (0.98-8.56) | 0.071^§^ |
| Esophageal cancer | 12 (16.91) | 4 (14.81) | 0.86 (0.25-2.93) | 0.999^§^ |
| Rectal cancer | 11 (15.50) | 2 (7.41) | 0.44 (0.09-2.11) | 0.505^§^ |
| Lung cancer | 7 (9.86) | 0 (0) | - | 0.185^§^ |
| Liver cancer | 4 (5.63) | 0 (0) | - | 0.573^§^ |
| Melanoma | 3 (4.23) | 0 (0) | - | 0.559^§^ |
| Others | 16 (22.54) | 5 (18.52) | 0.78 (0.26-2.39) | 0.665^ǂ^ |
| **Treatment lines of ICI, *n* (%)** | | | | |
| Neoadjuvant therapy | 19 (26.76) | 9 (33.33) | 1.37 (0.53-3.56) | 0.520^ǂ^ |
| Adjuvant therapy | 5 (7.04) | 0 (0) | - | 0.318^§^ |
| First-line therapy | 32 (45.07) | 13 (48.15) | 1.13 (0.47-2.75) | 0.785^ǂ^ |
| Second-line therapy | 8 (11.27) | 2 (7.41) | 0.63 (0.13-3.18) | 0.722^§^ |
| ≥Third-line therapy | 7 (9.86) | 3 (11.11) | 1.14 (0.27-4.78) | 0.999^§^ |
| **Type of ICIs, *n* (%)** | | | | |
| Sintilimab | 21 (29.58) | 11 (40.74) | 1.64 (0.65-4.11) | 0.292^ǂ^ |
| Toripalimab | 13 (18.30) | 4 (14.81) | 0.78 (0.23-2.63) | 0.774^§^ |
| Tislelizumab | 15 (21.13) | 2 (7.41) | 0.30 (0.06-1.41) | 0.141^§^ |
| Camrelizumab | 6 (8.45) | 3 (11.11) | 1.35 (0.31-5.88) | 0.703^§^ |
| Others | 16(22.54) | 7 (25.93) | 1.20 (0.43-3.35) | 0.723^ǂ^ |
| **Combined therapy, n (%)** | | | | |
| ICI monotherapy | 7 (9.86) | 7 (25.93) | 3.20 (1.00-10.23) | 0.053^§^ |
| Chemotherapy | 44 (61.97) | 16 (59.26) | 0.89 (0.36-2.21) | 0.806^ǂ^ |
| Targeted-therapy | 8 (11.27) | 1 (3.70) | 0.30 (0.04-2.55) | 0.437^§^ |
| Chemotherapy plus targeted therapy | 12 (16.90) | 3 (11.11) | 0.62 (0.16-2.37) | 0.754^§^ |
| **Cardiovascular risk factors, *n* (%)** | | | | |
| Hypertension | 18 (25.35) | 8 (29.63) | 1.24 (0.46-3.32) | 0.668^ǂ^ |
| Diabetes | 3 (4.23) | 3 (11.11) | 2.83 (0.54-15.00) | 0.342^§^ |
| Prior CV diseases | 11 (15.49) | 2 (7.41) | 0.44 (0.09-2.11) | 0.505^§^ |

Values are mean ± SD, n (%).

† Student’s t-test; ǂ Pearson Chi-square test; § Fisher’s exact test. For categorical variables, Pearson Chi-square test was used when all expected cell counts ≥ 5; Fisher’s exact test was applied when expected cell count < 5.

Abbreviations: ICI, immune checkpoint inhibitor; OR, odds ratio; CI, confidence interval; SD, standard deviation; CV, cardiovascular.
