# Supplementary material for: Integrated risk stratification for ICI-associated myocarditis: a baseline hematological profile and a combined ECG and enzymatic signature at onset
Source: Front Immunol. 2026 Mar 31;17:1762144. doi: 10.3389/fimmu.2026.1762144 (PMC13076345; doi:10.3389/fimmu.2026.1762144)
Supplement: Supplementary file 2 [file Table2.docx]

Supplementary Table 2. Comparative clinical characteristics at onset in mild versus severe ICI-associated myocarditis subgroups

| **Variables** | **Total (n = 98)** | **Mild myocarditis**  **(n = 71)** | **Severe myocarditis**  **(n = 27)** | **OR (95% CI)** | ***P* value** |
| --- | --- | --- | --- | --- | --- |
| **ICI cycles, median (IQR)** | 3(2.5) | 3 (2) | 2 (2) | - | 0.010^£^ |
| **Time from ICI initiation to onset of myocarditis (days), median (IQR)** | 48.5 (84.5) | 63 (80) | 33 (75) | - | 0.043^£^ |
| **Cardiac presentation, *n* (%)** | | | | | |
| Chest pain | 22 (22.45) | 4 (5.63) | 18 (66.67) | 33.50 (9.24-121.41) | < 0.001^ǂ^ |
| Dyspnea | 14 (14.29) | 2 (2.82) | 12 (44.44) | 27.60 (5.59-136.39) | < 0.001^§^ |
| Fatigue | 13 (13.27) | 2 (2.82) | 11 (40.74) | 23.72 (4.78-117.69) | < 0.001^§^ |
| **New onset ECG abnormalities, *n* (%)** | | | | | |
| T-wave changes | 24 (24.50) | 13 (18.31) | 11 (40.74) | 3.07 (1.16-8.13) | 0.021^ǂ^ |
| QT interval prolongation | 10 (10.20) | 3 (4.23) | 7 (25.93) | 7.93 (1.88-33.53) | 0.004^§^ |
| ST-T changes | 18 (18.37) | 13 (18.31) | 5 (18.52) | 1.01 (0.32-3.18) | 0.999^§^ |
| Complete/incomplete bundle branch block | 12 (12.24) | 3 (4.23) | 9 (33.33) | 11.33 (2.78-46.24) | < 0.001^§^ |
| Normal | 42 (42.86) | 39 (54.93) | 3 (11.11) | 0.10 (0.03-0.37) | < 0.001^ǂ^ |
| **New onset ECHO abnormalities, *n* (%)** | | | | | |
| Abnormal ECHO | 13 (13.27) | 6 (8.45) | 7 (25.93) | 3.79 (1.14-12.59) | 0.041^§^ |
| **New onset ECHO parameters, median (IQR)** | | | | | |
| LVEF (%) | 66 (8.00) | 67 (7.25) | 66 (16.50) | - | 0.287^£^ |
| **Laboratory cardiac biomarkers, *n* (%)** | | | | | |
| MYO, ng/mL | | | | | |
| Normal | 62 (63.27) | 47 (66.20) | 15 (55.56) | 0.64 (0.26-1.58) | 0.329^ǂ^ |
| ≤ 5× ULN | 20 (20.41) | 17 (23.94) | 3 (11.11) | 0.40 (0.11-1.48) | 0.159^ǂ^ |
| 5-10× ULN | 4 (4.08) | 3 (4.23) | 1 (3.70) | 0.87 (0.09-8.76) | 0.999^§^ |
| > 10× ULN | 12 (12.24) | 4 (5.63) | 8 (29.63) | 7.05 (1.92-25.98) | 0.003^§^ |
| HSTNI, ng/mL | | | | | |
| Normal | 41(41.84) | 35 (49.30) | 6 (22.22) | 0.29 (0.11-0.82) | 0.015^ǂ^ |
| ≤ 5× ULN | 17 (17.35) | 14 (19.72) | 3 (11.11) | 0.51 (0.13-1.93) | 0.385^§^ |
| 5-10× ULN | 12 (12.24) | 8 (11.27) | 4 (14.81) | 1.37 (0.38-4.98) | 0.732^§^ |
| > 10× ULN | 28 (28.57) | 14 (19.72) | 14 (51.85) | 4.39 (1.69-11.39) | 0.002^ǂ^ |
| CK, U/L | | | | | |
| Normal | 51 (52.04) | 38 (53.52) | 13 (48.15) | 0.81 (0.33-1.96) | 0.634^ǂ^ |
| ≤ 5× ULN | 25 (25.51) | 23 (32.39) | 2 (7.41) | 0.17 (0.04-0.77) | 0.011^ǂ^ |
| 5-10× ULN | 7 (7.14) | 5 (7.04) | 2 (7.41) | 1.06 (0.19-5.80) | 0.999^§^ |
| > 10× ULN | 15 (15.31) | 5 (7.04) | 10 (37.04) | 7.77 (2.34-25.74) | 0.001^§^ |
| CK-MB, U/L | | | | | |
| Normal | 61(62.24) | 47 (66.20) | 14 (51.85) | 0.55 (0.22-1.35) | 0.191^ǂ^ |
| ≤ 5× ULN | 25 (25.51) | 19 (26.76) | 6 (22.22) | 0.78 (0.27-2.23) | 0.645^ǂ^ |
| > 5× ULN | 12 (12.24) | 5 (7.04) | 7 (25.93) | 4.62 (1.32-16.16) | 0.017^§^ |
| α-HBD, U/L | | | | | |
| Normal | 60 (61.22) | 47 (66.20) | 13 (48.15) | 0.47 (0.19-1.17) | 0.101^ǂ^ |
| ≤ 5× ULN | 34 (34.69) | 24 (33.80) | 10 (37.04) | 1.15 (0.46-2.90) | 0.764^ǂ^ |
| > 5× ULN | 4 (4.08) | 0 (0) | 4 (14.81) | - | 0.005^§^ |
| LDH, U/L | | | | | |
| Normal | 36 (36.73) | 31 (43.66) | 5 (18.52) | 0.29 (0.10-0.86) | 0.021^ǂ^ |
| ≤ 5× ULN | 48 (48.98) | 34 (47.89) | 14 (51.85) | 1.17 (0.48-2.85) | 0.726^ǂ^ |
| > 5× ULN | 5 (5.10) | 1 (1.41) | 4 (14.81) | 12.17 (1.29-114.50) | 0.020^§^ |
| AKP, U/L | | | | | |
| Normal | 92 (93.88) | 67 (94.37) | 25 (92.59) | 0.75 (0.13-4.33) | 0.666^§^ |
| ≤ 5× ULN | 6 (6.12) | 4 (5.63) | 2 (7.41) | 1.34 (0.23-7.78) | 0.666^§^ |
| **Other laboratory results, mean ± SD / median (IQR)** | | | | | |
| WBC, ×10^9^/L | 6.77 (4.77) | 6.47 (4.35) | 7.87 (5.65) | - | 0.345^£^ |
| Hemoglobin, g/L | 106.93 ± 26.24 | 108.3 ± 26.11 | 102.96 ± 26.72 | - | 0.221^£^ |
| Platelets, ×10^9^/L | 219.50 (109.75) | 222.00 (110.00) | 217.00 (126.50) | - | 0.735^£^ |
| ALT, U/L | 33.58 (50.20) | 25.85 (39.51) | 58.84 (97.69) | - | 0.002^£^ |
| Creatinine, μmol/L | 75.24 (41.13) | 77.00 (41.60) | 70.20 (40.30) | - | 0.523^£^ |
| Albumin, g/L | 37.67 ± 5.76 | 38.38 ± 5.67 | 35.68 ± 5.69 | - | 0.039^£^ |
| D-dimer, μg/mL | 1.63 (3.60) | 1.43 (2.35) | 2.15 (5.53) | - | 0.098^£^ |
| hsCRP, mg/L | 14.52(59.70) | 14.52 (56.30) | 14.80 (126.54) | - | 0.621^£^ |
| **Concurrent irAEs, *n* (%)** | | | | | |
| Myositis | 7 (7.14) | 1 (1.41) | 6 (22.22) | 20.00 (2.28-175.59) | 0.002^§^ |
| Pneumonia | 7 (7.14) | 0 (0) | 7 (25.93) | - | ＜0.001^§^ |
| Hepatitis | 37 (37.75) | 22 (30.99) | 15 (55.56) | 2.78 (1.12-6.92) | 0.025^ǂ^ |
| Hypothyroidism | 12 (12.24) | 9 (12.68) | 3 (11.11) | 0.86 (0.22-3.45) | 0.999^§^ |

Values are mean ± SD, median (IQR) or n (%).

£ Mann-Whitney U test; ǂ Pearson Chi-square test; § Fisher's exact test. For categorical variables, Pearson Chi-square test was used when all expected cell counts ≥ 5; Fisher's exact test was applied when expected cell count < 5.

Abbreviations: ICI, immune checkpoint inhibitor; OR, odds ratio; CI, confidence interval; IQR, interquartile range; ECG, electrocardiogram; ECHO, echocardiograph. LVEF, left ventricular ejection fraction; MYO, myoglobin; HSTNI, high-sensitivity troponin I; CK, creatine kinase; CK-MB, creatine kinase MB; α-HBD, α-hydroxybutyrate dehydrogenase; LDH, lactate dehydrogenase; AKP, alkaline phosphatase; ULN, upper limit of normal; SD, standard deviation; WBC, white blood cell count; ALT, alanine aminotransferase; hsCRP, high sensitivity C-reactive protein; irAE, immune-related adverse event.
